# Supplementary material for: Physiologically relevant reconstitution of iron-sulfur cluster biosynthesis uncovers persulfide-processing functions of ferredoxin-2 and frataxin
Source: Nat Commun. 2019 Aug 8;10:3566. doi: 10.1038/s41467-019-11470-9 (PMC6687725; doi:10.1038/s41467-019-11470-9)
Supplement: Supplementary file 1 — Supplementary information [file 41467_2019_11470_MOESM1_ESM.pdf]

Supplementary Information

Physiologically relevant reconstitution of iron-sulfur cluster biosynthesis uncovers persulfide-processing functions of ferredoxin-2 and frataxin

Gervason, Larkem, Ben Mansour *et al.*

Supplementary Figures

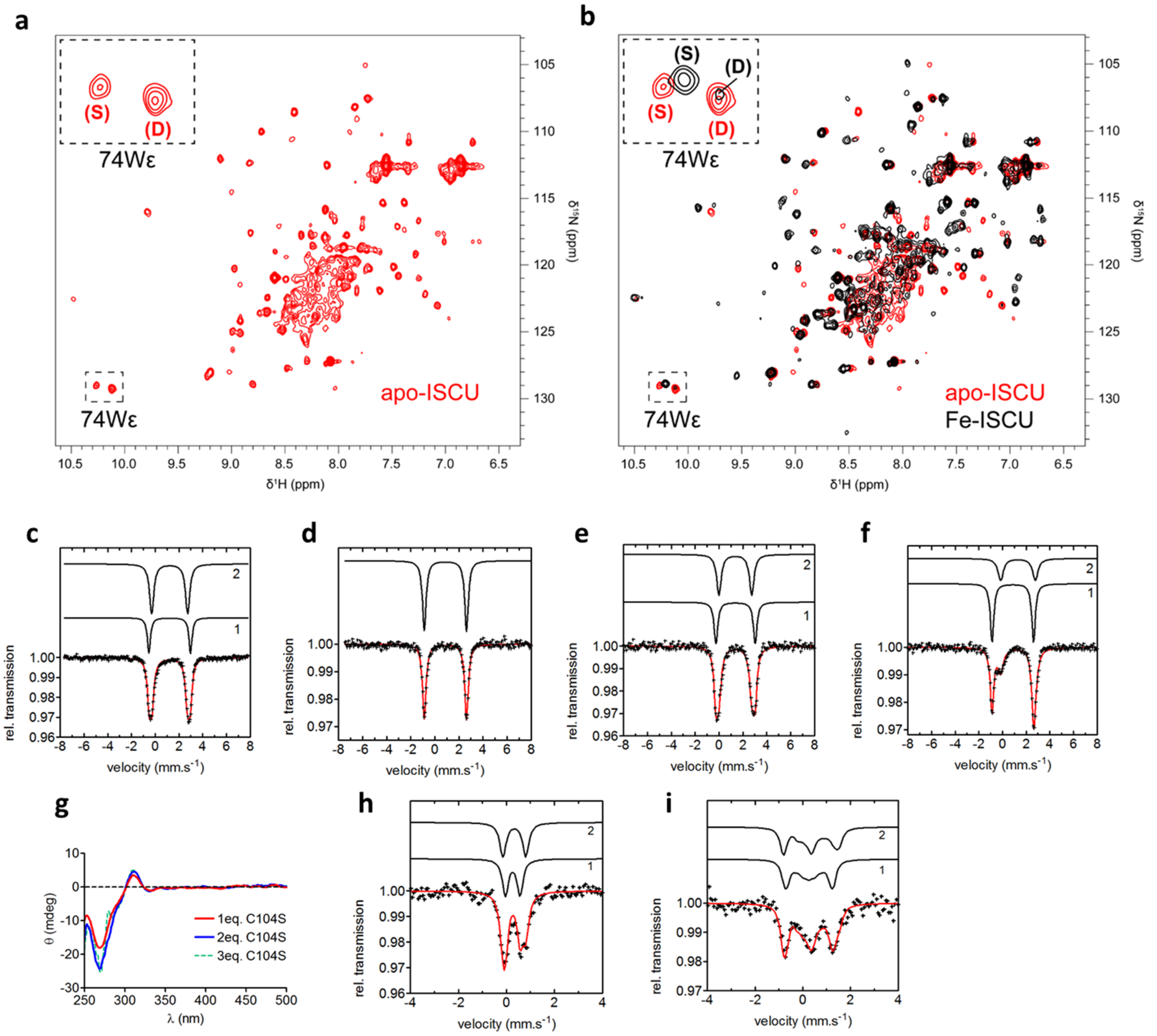

Supplementary Figure 1: Characterization of Fe-ISCU and its Fe-S reconstituted form

(**a**, **b**) ^{1}H-^{15}N SOFAST-HMQC NMR spectra of apo-ISCU (100 μM) in red (**a**, **b**) and apo-ISCU incubated with one equivalent of iron in black (**b**). The insets show close-ups of the W74 Hε-Nε signals assigned to structured (S) and disordered (D) conformations. Spectra were acquired at 293 K and 800 MHz ^{1}H resonance frequency. (**c**) Mössbauer spectrum of ferrous iron in buffer T. (**d**) Mössbauer spectrum of ISCU WT incubated with 0.6 equivalent of iron. (**e-f**) Mössbauer spectra of the ISCU mutants H103A (**e**) and C104S (**f**) incubated with one equivalent of iron. (**g**) Titration of iron by the C104S mutant monitored by CD. Increasing amounts of the C104S mutant (100, 200 and 300 μM) were incubated with a fixed concentration of iron (100 μM). At 300 μM the protein starts to precipitate which precludes to complete the titration. (**h**,**i**) Applied field Mössbauer spectra of Fe-ISCU reconstituted with a Fe-S cluster. The spectra were recorded at T = 4.2 K in an external field B = 0.1 T (**h**) and B = 5 T (**i**). The red solid line represents the best fits of the data achieved using the components displayed as black solid lines. (See **Supplementary Table 1** for the respective parameters of each component).

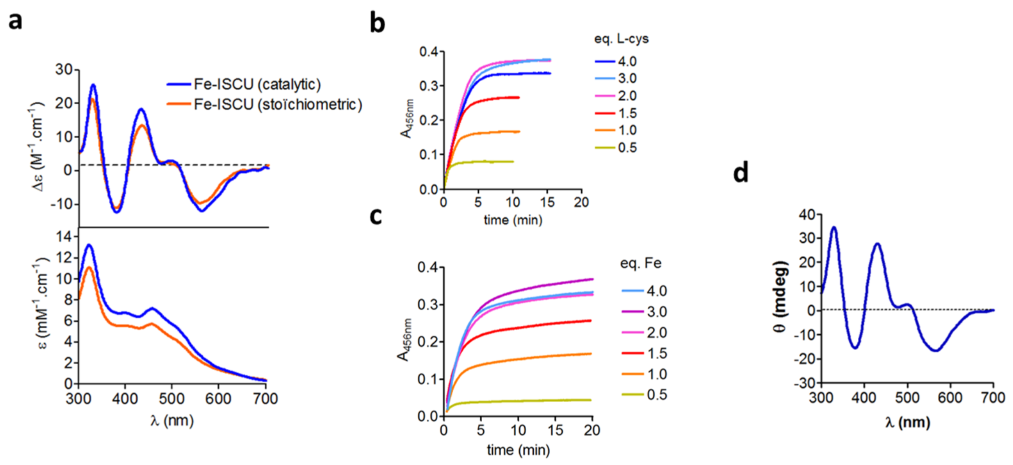

Supplementary Figure 2: Characterization of Fe-S cluster formation in the NIAUF complex and iron and L-cysteine titrations

(**a**) UV-visible and CD spectra of ISCU reconstituted under catalytic (free ISCU) and stoichiometric (ISCU in the NIAUF complex) conditions. The spectra are displayed in molar absorption units based on the absorption coefficients of the [2Fe2S] in free ISCU and in the NIAUF complex. (**b**) Fe-S cluster reconstitution assays under catalytic conditions at various concentrations of L-cysteine as indicated in molar equivalents with regard to ISCU and using 2 equivalents of iron. (**c**) Fe-S cluster reconstitution assays under catalytic conditions at various concentrations of iron as indicated in molar equivalents as regards to ISCU and using 2 equivalents of L-cysteine. (**d**) CD spectrum of Fe-S cluster reconstitution by Fe-ISCU performed under catalytic conditions in the absence of FXN, with one equivalent of L-cysteine.

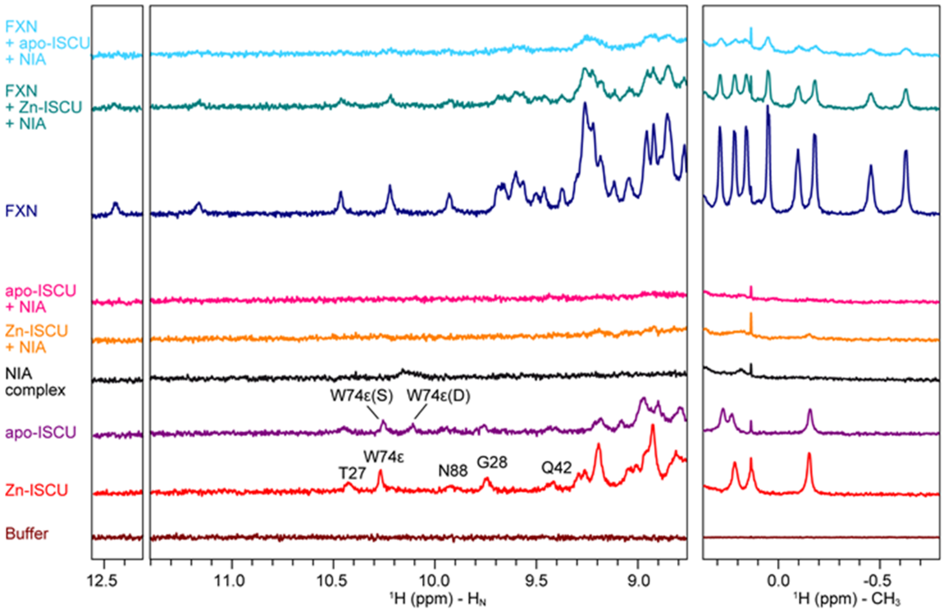

Supplementary Figure 3: Formation of the NIAUF complex with apo-ISCU

^{1}H NMR spectra excerpts zoomed in the amide (H_N) (left and middle panel) and methyl (CH_3) (right panel) regions of Zn-ISCU, apo-ISCU, the NIA complex and FXN, and mixtures of NIA complex with Zn-ISCU or apo-ISCU in a 1:1 molar ratio, FXN with NIA complex and Zn-ISCU, or NIA complex and apo-ISCU, in a 1:1:1 ratio. Intensities in the methyl region were scaled down relative to the amide region. Spectra were acquired at a temperature of 293 K and at 800 MHz ^{1}H resonance frequency. The assignments of the two signals from W74-Hε in apo-ISCU corresponding to the S and D conformational states are indicated as well as those of ISCU amide signals with $\delta^{1}H > 9.4$ ppm.

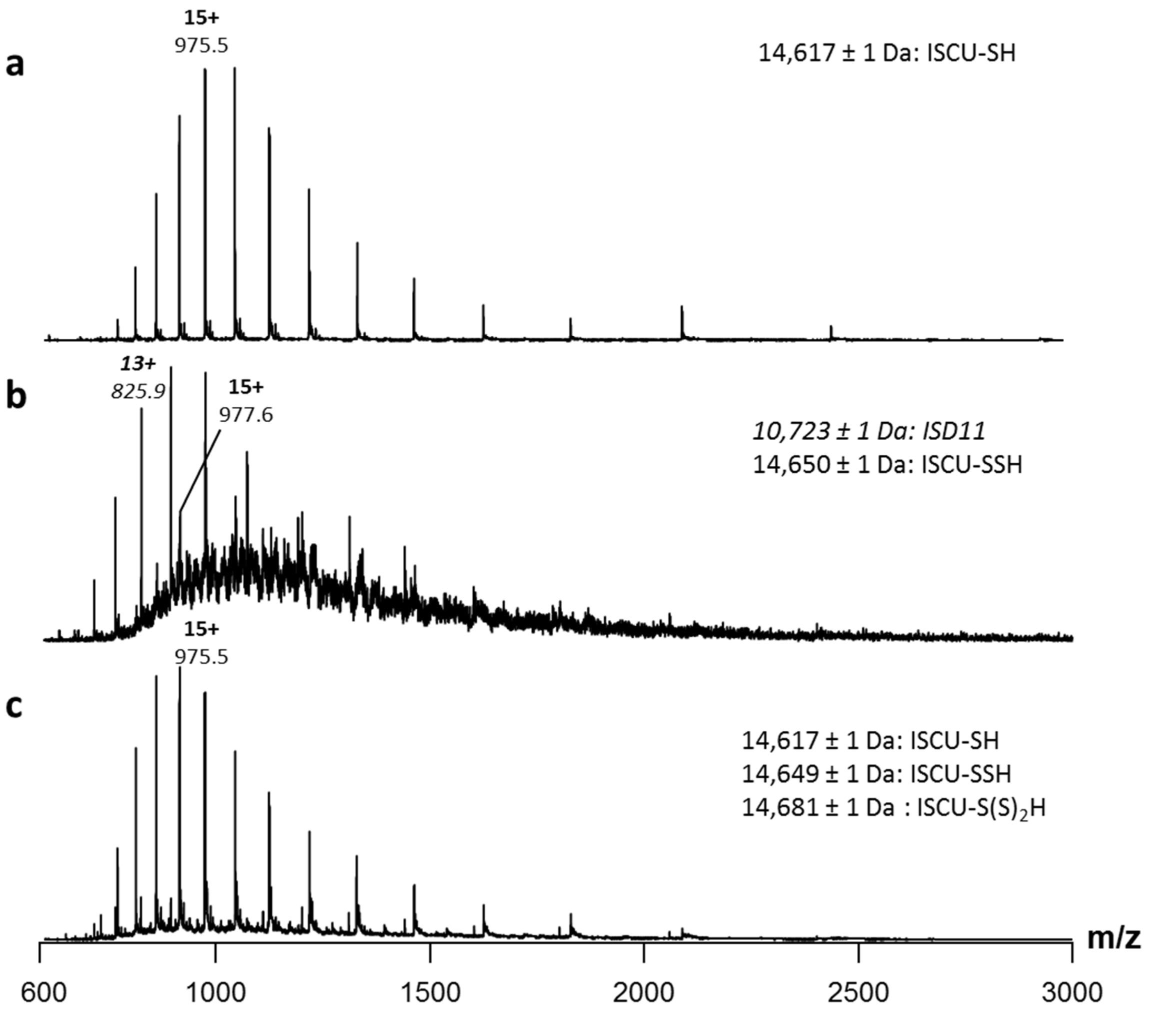

Supplementary Figure 4: Raw mass spectra of persulfidation states of ISCU

Raw mass spectra corresponding to the deconvoluted spectra of (**a**) Fig. 3c, (**b**) Fig. 3d and (**c**) Fig. 3e. See **Table 3** for the theoretical and experimental masses.

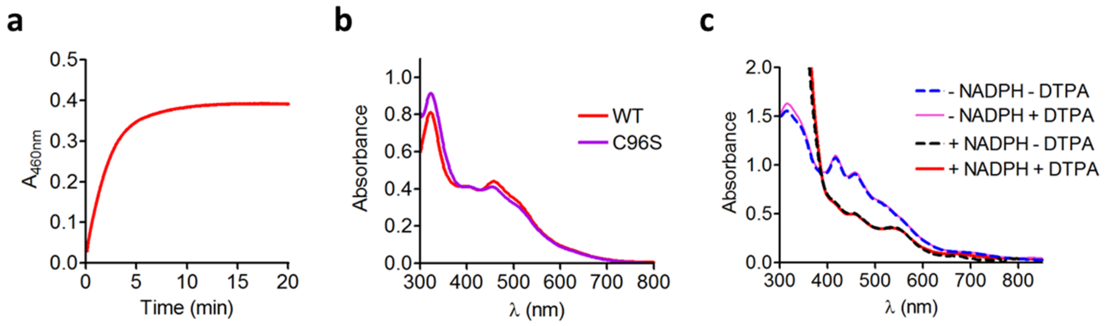

Supplementary Figure 5: Properties of the C96S ISCU mutant and effect of DTPA on FDX2

(**a**) Kinetic of Fe-S cluster assembly under catalytic conditions with the C96S mutant of ISCU. (**b**) UV-visible spectrum of Fe-S cluster reconstituted C96S ISCU as performed in (a). (**c**) UV-visible spectra of mixture containing oxidized FDX2 and FDXR incubated with (pink line) and without (dashed blue line) DTPA and upon addition of NADPH in reactions missing (dashed black line) and containing (red line) DTPA. DTPA does not alter the UV-visible spectrum of FDX2, indicating that it is not able to chelate the iron of the [2Fe2S] cluster of FDX2. Upon addition of NADPH, the spectrum of FDX2 is modified in the same way both in the absence and presence of DTPA, which indicates that the [2Fe2S] cluster is reduced by FDXR and that DTPA does not interfere with this reaction.

Supplementary Tables

Supplementary Table 1: Mössbauer parameters of samples from this study

Sample	Eq. Fe	T (K)	B (T)	component	Area (%)	δ (mm/s)	ΔE_Q (mm/s)	Γ (mm/s)	η (mm/s)
**WT ISCU**	1	77	0	1	85	0.89	3.51	0.32	
				2	15	1.12	2.77	0.6	
**Fe**	-	77	0	1	34	1.22	3.5	0.31	
				2	66	1.22	3.03	0.43	
**WT ISCU**	0.6	77	0	1	100	0.88	3.51	0.35	
**H103A ISCU**	1	77	0	1	52	1.23	3.43	0.40	
				2	48	1.23	2.84	0.45	
**C104S ISCU**	1	77	0	1	63	0.87	3.51	0.35	
				2	37	1.23	3.15	0.65	
**Fe-S reconstituted WT ISCU**		77	0	1	50	0.26	0.61	0.28	
				2	50	0.33	0.95	0.34	
		4.2	0.1	1	50	0.26	0.61	0.29	1 (-0.3)
				2	50	0.33	0.95	0.31	0 (+0.3)
		4.2	5	1	50	0.26	0.61	0.29	1 (-0.3)
				2	50	0.33	0.95	0.31	0 (+0.3)

Supplementary Table 2: Selected Mössbauer parameters of ferrous iron center in proteins with various combinations of O, N and S coordinating atoms

Proteins	Environment	δ (mm/s)	ΔE_Q (mm/s)	reference
Mus musculus ISCU		0.89	3.51	this work
*D. desulfuricans SOR center I (*Desulfoferrodoxin)	4S	0.66	2.90	[1]
C. pasteurianum Rubredoxin	4S	0.70	- 3.25	[2]
C. pasteurianum Rubredoxin C42S	3S, 1O	0.79	2.95<br>- 3.27	[3]
Horse Alcohol Dehydrogenase	2S, 1N, 1N/O	0.83	3.84	[4]
P. aeruginosa Azurin	1S, 2N, 1O	0.92	3.19	[5]
*D. desulfuricans SOR center II (*Desulfoferrodoxin)	1S, 4N	1.04	2.87	[6]
Soybean Lipoxygenase	3N, 20	1.10	3.08	[7]
E. coli FUR site 2	3N, 2O	1.19	3.47	[8]
H. sapiens Tyrosine Hydroxylase 1	6N/0	1.24	2.68	[9]

Supplementary Table 3: List of theoretical and experimental masses of ISCU persulfidation states determined from spectra presented in **Fig. 3c-e** and **Supplementary Fig. 4**.

Species	Theoretical mass (Da)	Experimental mass (Da)	Error (ppm)
ISCU-SH	14,618	14,617 ± 1	68
ISCU-SSH	14,650	14,650 ± 1	-
ISCU-$S(S)_2H$	14,682	14,681 ± 1	68

Supplementary Notes

Supplementary Note 1: Mössbauer analysis of WT, C104S and H103A ISCU proteins incubated with iron

Mössbauer spectroscopy provides direct information on the nature of the atoms coordinating an iron ion. There is a linear correlation between the gain in isomer shift and the number of electronegative ligands coordinating the iron center: the more electronegative the ligand, the higher the isomer shift.[10] Fe(II) centers in proteins with 4 sulfur ligands typically exhibit an isomer shift in the range of 0.60 – 0.70 $mm.s^{-1}$. Adding more electronegative ligands such as nitrogen and oxygen ligands leads to an increase of the isomer shift with values higher than 1.2 $mm.s^{-1}$ for Fe(II) centers with six oxygen and nitrogen and no sulfur coordination (see **Supplementary Table 2** for selected Fe(II) centers).

Based on these empiric correlations, the isomer shifts of component 1 and 2 in the sample of WT ISCU are both consistent with high spin Fe(II) centers in mixed environments but with one or two sulfur ligands and two or three N/O ligands for component 1 and five or six N/O atoms but no sulfur for component 2 (**Supplementary Table 1 and 2**). The proportions of two components detected by Mössbauer appear correlated with those of the S and D states.

The Mössbauer spectrum of the H103A mutant reveals the presence of two components with parameters similar to those of free iron (**Supplementary Fig. 1e** and **Supplementary Table 1**). This indicates that the H103A mutation precludes iron binding in the cysteinyl site, leaving iron free or promiscuously bound to ISCU. Interestingly the minor species identified in WT ISCU (component 2) does not seem to be present in H103A mutant, this suggests that H103 may be a ligand in component 2.

The C104S mutant incubated with 1 equivalent of iron reveals the presence of two components. A major species (65 %) with Mössbauer parameters identical to those of iron in the assembly site of the WT protein and a minor component (45 %) with parameters similar to those of free iron (**Supplementary Fig. 1f** and **Supplementary Table 1**). The lower proportion of iron in the assembly site compared to WT ISCU suggests that either a proportion of the C104S mutant is compromised for iron binding or that the C104S mutation lowers the affinity of iron for the assembly site. By increasing the ratio of the C104S mutant over iron, keeping the concentration of iron constant, we found that the CD signal increases reaching similar intensity as the WT (**Supplementary Fig. 1g**). This indicates that the C104S

mutant has lower affinity for iron than WT. The absence of the minor species detected in WT ISCU (component 2) suggests that C104 is also required for binding of iron to the D state of apo-ISCU, although not ligating the iron.

Supplementary Note 2: Analysis of Mössbauer data of Fe-S reconstituted ISCU

The best fit of the Mössbauer data was obtained with two components in a 50:50 ratio. The values of the isomer shifts are in the range of 0.25 to 0.35 $mm.s^{-1}$ which is characteristic of oxidized iron in a $[2Fe2S]^{2+}$ cluster (**Fig. 2c, Supplementary Fig. 1h, 1i** and **Supplementary Table 1**). The 50:50 ratio of components 1 and 2 further support the presence of a dinuclear center. The presence of [4Fe4S] clusters can be excluded based on the isomer shifts, since values in the range of 0.4 to 0.9 $mm.s^{-1}$ are expected for a [4Fe4S] cluster.[11] Moreover, the best fits of the field dependent Mössbauer spectra were achieved by considering that the Fe-S cluster is diamagnetic species.[11] This indicates that the resulting spin state of the [2Fe2S] cluster is S = 0 which is consistent with two antiferromagnetically coupled Fe^{3+} ions and thus confirms that the [2Fe2S] cluster is in its oxidized state with an overall +2 charge. Moreover, the iron ions in the $[2Fe2S]^{2+}$ cluster are not equivalent, they exhibit different set of parameters which point to different environments. The isomer shifts suggest that the Fe^{3+} ion in site 1 is coordinated by four sulfur atoms and the iron in site 2 by two or three sulfurs and one or two N/O ligand(s).[11,12]

Supplementary Note 3: Formation of complexes with apo-ISCU

The 1D ^{1}H NMR spectra of apo-ISCU and Zn-ISCU show a complete disappearance of resonances upon mixing with one molar equivalent of the NIA complex (see **Supplementary Fig. 3**). As the size of a protein increases, its rotational motion in solution is slowed down and the NMR signals widths increase. The signals of the large NIA complex are broad to such a point that they become nearly undetectable. The apparent disappearance of the signals of ISCU in the presence of the NIA complex is attributed to complete broadening of the signals and thus provides evidence for complex formation.

Upon mixing FXN with the NIAU complex containing either apo-ISCU or Zn-ISCU, the characteristic peaks of FXN are strongly diminished which points to association of FXN to the NIAU complex. However, as the peaks do not totally disappear, some free FXN remains. At a 1:1 molar ratio, ~ 10 % and 35 % of free FXN signals are still observed for the complexes formed with apo-ISCU and Zn-ISCU, respectively. This points to affinities of

FXN in the micromolar range for both complexes and a higher affinity for the complex containing apo-ISCU.

Supplementary References

1 Tavares, P., Ravi, N., Moura, J. J. G., Legall, J., Huang, Y. H., Crouse, B. R., Johnson, M. K., Huynh, B. H. ,Moura, I. Spectroscopic Properties of Desulfoferrodoxin from Desulfovibrio-Desulfuricans (Atcc-27774). *Journal of Biological Chemistry* (1994) **269**, 10504-10510

2 Phillips, W. D., Poe, M., Weiher, J. F., Mcdonald, C. C. ,Lovenberg, W. Proton Magnetic Resonance, Magnetic Susceptibility and Mossbauer Studies of Clostridium-Pasteurianum Rubredoxin. *Nature* (1970) **227**, 574-+

3 Yoo, S. J., Meyer, J., Achim, C., Peterson, J., Hendrich, M. P. ,Munck, E. Mossbauer, EPR, and MCD studies of the C9S and C42S variants of Clostridium pasteurianum rubredoxin and MCD studies of the wild-type protein. *Journal of Biological Inorganic Chemistry* (2000) **5**, 475-487

4 Bill, E., Haas, C., Ding, X. Q., Maret, W., Winkler, H., Trautwein, A. X. ,Zeppezauer, M. Fe(Ii)-Substituted Horse Liver Alcohol-Dehydrogenase, a Model for Non-Heme Iron Enzymes - Various States of Iron-Dioxygen Interaction Investigated by Mossbauer and Epr Spectroscopy. *Eur J Biochem* (1989) **180**, 111-121

5 McLaughlin, M. P., Retegan, M., Bill, E., Payne, T. M., Shafaat, H. S., Pena, S., Sudhamsu, J., Ensign, A. A., Crane, B. R., Neese, F. ,Holland, P. L. Azurin as a Protein Scaffold for a Low-coordinate Nonheme Iron Site with a Small-molecule Binding Pocket. *Journal of the American Chemical Society* (2012) **134**, 19746-19757

6 Moura, I., Tavares, P., Moura, J. J. G., Ravi, N., Huynh, B. H., Liu, M. Y. ,Legall, J. Purification and Characterization of Desulfoferrodoxin - a Novel Protein from Desulfovibrio-Desulfuricans (Atcc-27774) and from Desulfovibrio-Vulgaris (Strain Hildenborough) That Contains a Distorted Rubredoxin Center and a Mononuclear Ferrous Center. *Journal of Biological Chemistry* (1990) **265**, 21596-21602

7 Dunham, W. R., Carroll, R. T., Thompson, J. F., Sands, R. H. ,Funk, M. O. The Initial Characterization of the Iron Environment in Lipoxygenase by Mossbauer-Spectroscopy. *Eur J Biochem* (1990) **190**, 611-617

8 Jacquamet, L., Dole, F., Jeandey, C., Oddou, J. L., Perret, E., Le Pape, L., Aberdam, D., Hazemann, J. L., Michaud-Soret, I. ,Latour, J. M. First spectroscopic characterization of Fe-II-Fur, the physiological active form of the Fur protein. *Journal of the American Chemical Society* (2000) **122**, 394-395

9 Schunemann, V., Meier, C., Meyer-Klaucke, W., Winkler, H., Trautwein, A. X., Knappskog, P. M., Toska, K. ,Haavik, J. Iron coordination geometry in full-length, truncated, and dehydrated forms of human tyrosine hydroxylase studied by Mossbauer and X-ray absorption spectroscopy. *Journal of Biological Inorganic Chemistry* (1999) **4**, 223-231

10 Gütlich, P., Bill, E. ,Trautwein, A. X. *Mössbauer Spectroscopy and Transition Metal Chemistry. Fundamentals and Applications*. 2 edn, (Springer-Verlag, 2011).

11 Pandelia, M. E., Lanz, N. D., Booker, S. J. ,Krebs, C. Mossbauer spectroscopy of Fe/S proteins. *Bba-Mol Cell Res* (2015) **1853**, 1395-1405

12 Garcia-Serres, R., Clémancey, M., Latour, J.-M. ,Blondin, G. Contribution of Mössbauer spectroscopy to the investigation of Fe/S biogenesis. *JBIC Journal of Biological Inorganic Chemistry* (2018) **23**, 635-644
